# Data accuracy in the Ontario birth Registry: a chart re-abstraction study

**DOI:** 10.1186/s12913-019-4825-3

**Published:** 2019-12-27

**Authors:** Sandra Dunn, Andrea Lanes, Ann E. Sprague, Deshayne B. Fell, Deborah Weiss, Jessica Reszel, Monica Taljaard, Elizabeth K. Darling, Ian D. Graham, Jeremy M. Grimshaw, JoAnn Harrold, Graeme N. Smith, Wendy Peterson, Mark Walker

**Affiliations:** 1 Better Outcomes Registry & Network , Ottawa, Ontario Canada; 2 Children’s Hospital of Eastern Ontario Research Institute, Ottawa, Ontario Canada; 3 University of Ottawa, Ottawa, Ontario Canada; 4 The Ottawa Hospital Research Institute, Ottawa, Ontario Canada; 5 McMaster University, Hamilton, Ontario Canada; 6 Children’s Hospital of Eastern Ontario, Ottawa, Ontario Canada; 7 The Ottawa Hospital, Ottawa, Ontario Canada; 8 Kingston General Hospital and Queen’s University, Kingston, Ontario Canada

**Keywords:** Data accuracy, Re-abstraction, Data quality assessment, BORN Ontario

## Abstract

**Background:**

Ontario’s birth Registry (BORN) was established in 2009 to collect, interpret, and share critical data about pregnancy, birth and the early childhood period to facilitate and improve the provision of healthcare. Since the use of routinely-collected health data has been prioritized internationally by governments and funding agencies to improve patient care, support health system planning, and facilitate epidemiological surveillance and research, high quality data is essential. The purpose of this study was to verify the accuracy of a selection of data elements that are entered in the Registry.

**Methods:**

Data quality was assessed by comparing data re-abstracted from patient records to data entered into the Ontario birth Registry. A purposive sample of 10 hospitals representative of hospitals in Ontario based on level of care, birth volume and geography was selected and a random sample of 100 linked mother and newborn charts were audited for each site. Data for 29 data elements were compared to the corresponding data entered in the Ontario birth Registry using percent agreement, kappa statistics for categorical data elements and intra-class correlation coefficients (ICCs) for continuous data elements.

**Results:**

Agreement ranged from 56.9 to 99.8%, but 76% of the data elements (22 of 29) had greater than 90% agreement. There was almost perfect (kappa 0.81–0.99) or substantial (kappa 0.61–0.80) agreement for 12 of the categorical elements. Six elements showed fair-to-moderate agreement (kappa <0.60). We found moderate-to-excellent agreement for four continuous data elements (ICC >0.50).

**Conclusion:**

Overall, the data elements we evaluated in the birth Registry were found to have good agreement with data from the patients’ charts. Data elements that showed moderate kappa or low ICC require further investigation.

## Background

Ontario’s province-wide birth Registry (Better Outcomes Registry & Network [BORN Ontario]) was established in 2009 to collect, interpret, and share critical data about pregnancy, birth and the early childhood period. As a prescribed Registry under provincial privacy legislation, BORN Ontario safeguards data while making information available to facilitate and improve the provision of healthcare.

The BORN Registry, an Internet-based data collection system, was launched in January 2012, but historical perinatal data are available from 2006 from a pre-existing data collection platform. Sourced from hospitals, labs, midwifery practice groups and clinical programs, the data are either manually entered by hospital staff or uploaded directly from hospitals’ electronic medical records. The scope of the data spans the antepartum, intrapartum, and postpartum periods and includes information on maternal demographics and health behaviours, pre-existing maternal health problems, pregnancy and obstetric complications, intrapartum interventions, and birth and newborn outcomes. These data are captured at the time of birth from medical records, clinical forms, and patient interviews for all hospitals births as well as home and birth centre births in Ontario. With nearly 40% of all live births in Canada occurring in Ontario (36.7% in 2016) [1], this database is a rich source of perinatal information for a large proportion of the births in Canada. Data from the BORN Registry are widely used to facilitate care, support clinicians, inform policy makers, and conduct research to increase knowledge about optimal care [2–16].

This relatively new system uses a complex method of collecting data at the different points during pregnancy, birth and into childhood, often collecting the same data element multiple times throughout the course of care. These data are then brought together to form a unified maternal-newborn record using robust linking and matching algorithms. Duplicated data elements from multiple care encounters are also aggregated through a complex set of decision rules into the final unified record in the Registry. Each contributing site has access to their own data through a robust and secure reporting portal and BORN Ontario reports on outcomes aggregated at the provincial level at regular intervals [17–21].

Since the use of routinely-collected health data has been prioritized internationally by governments and funding agencies to improve patient care, support health system planning and health care efficiency, facilitate epidemiological surveillance, and transform research, access to high quality data is essential [22, 23]. Formal processes for regular data validation, quality checks, and training for individuals entering and using the data have been implemented to support a high level of data quality [24, 25]. However, as with any administrative dataset, these data may be vulnerable to random and systematic errors due to incomplete or illegible documentation in the patient health record, human error during manual data entry, electronic health record upload errors, unclear definitions, or inadequately trained personnel [24]. Given the complex nature of the data collected in the Registry, the objective of our study was to assess the accuracy of a subset of core data elements by conducting a complete reabstraction audit comparing data entered into the BORN Registy with data from the patient health record. This paper reports the results of the study.

## Methods

### Theoretical framework

**Fig. 1:**
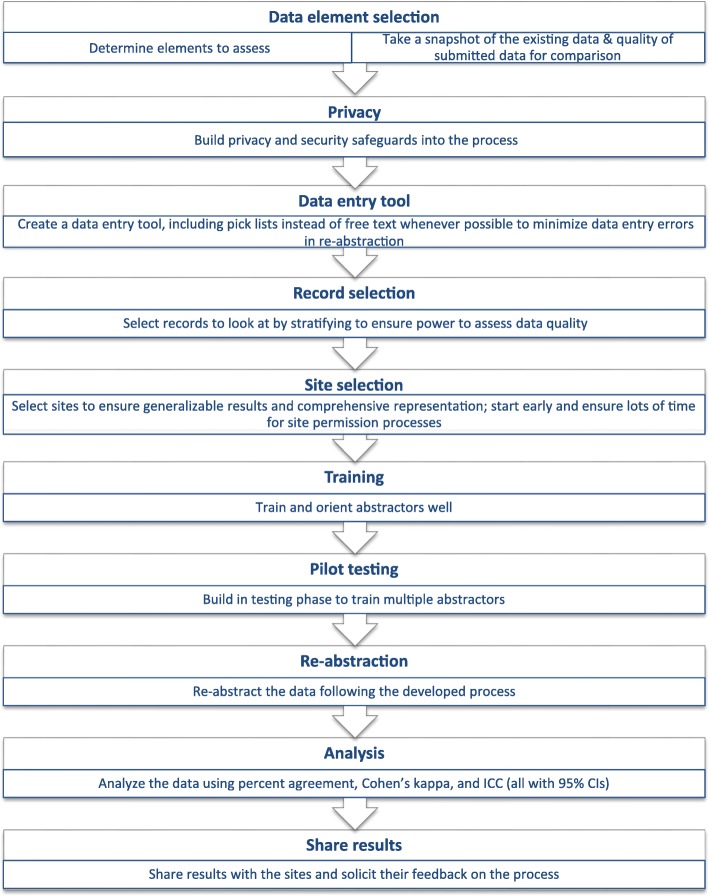
BORN re-abstraction process

We used the data quality framework adopted by BORN Ontario which is based on five dimensions: timeliness, accuracy (validity), comparability (reliability), usability and relevance to guide this study [25, 26]. Additionally, we followed similar methods to those described by other re-abstraction studies such as the Data Quality Assessment of the Niday Perinatal Database [24], the Canadian Institute for Health Information (CIHI) Data Re-Abstraction Study (2015–2016) [27], and the British Columbia Perinatal Data Registry re-abstraction study [28]. The re-abstraction process is outlined in Fig. 1.

### Ethics and privacy issues

This re-abstraction study was a quality assurance project, and therefore exempt from Research Ethics Board review under article 2.5 of the Tri-council Policy Statement [29]. Hospital participation was voluntary, and confidentiality of patient information and privacy of participating hospitals was maintained. The auditors re-entered data from the patient records that had previously been entered by the hospital personnel into the Registry. The re-abstracted electronic data were entered into a secure online data capture system, Research Electronic Data Capture (REDCap) [30], and then stored and analyzed on a secure network drive. All data were aggregated, and findings were anonymized.

### Site recruitment, record selection, and creation of a re-abstraction tool

We recruited participant hospitals from different health regions in the province. We aimed to have representation from all levels of care, geographic regions, and different data entry methods (i.e., manual entry versus electronic upload). Selected sites were provided with information about the project and invited to participate. For those that agreed to participate, the appropriate permissions to undertake a data quality assessment and allow our data auditors access to patient charts were obtained.

We selected a subset of data elements (*n* = 29) from the Registry for re-abstraction based on the following criteria: a) data element was used in the computation of key performance indicators in BORN Ontario’s Maternal Newborn Dashboard [5]; and b) data element was prioritized for validation by the BORN Data Quality Team based on operational requirements for reporting and research (e.g., maternal height and weight used to calculate Body Mass Index (BMI) and maternal smoking).

We produced a computer-generated random sample of 100 maternal chart numbers (and linked baby records) for each of the 10 participating sites from existing records that had already been entered into the Registry database in 2014–15. At the time of the study there were 96 hospitals in Ontario providing maternal-newborn care with approximately 140,000 births annually. We targeted 1000 records to provide a reasonable sample size for calculating measures of agreement based on the prevalence rates of the data elements under review. This sample size was primarily based on feasibility; however, we calculated that, for an expected kappa of 0.85, a total of 1000 records yields a two-sided 95% confidence interval with a total width of 0.111 (i.e., a lower limit of 0.80) if the prevalence is 90% using Fleiss’s large-sample formula. If the prevalence is lower, say 50%, the total width narrows to 0.065 (lower limit of 0.82). These margins of error were considered acceptable by the team.

A list of the chart numbers for reabstraction was sent via a secure messaging system to the hospital liaison for each site, to obtain access to the charts for the auditors. A data abstraction tool was prepared using REDCap [31]. REDCap allowed for data validation and cleaner data structuring of abstracted data, but also for the creation of a screen specific to a given entered record.

### Abstractor orientation

Two experienced auditors conducted the on-site audits. As a part of the auditor training, we created a detailed definition for each of the data elements to be re-abstracted, based on the current documentation available to those entering Registry data. We then consulted with clinical experts to determine a hierarchy of chart documentation, so that the abstractors could choose between conflicting information that may be recorded in different sections of the chart.

At each site, the auditors received basic, standardized orientation to the hospital-specific data entry systems and practices, and were trained to obtain information from the same sources used for the original data entry (e.g., the admission record, the provincial antenatal record, the labour and birth record, the discharge summary, lab results, etc.). Each auditor was given access to an online REDCap file with pre-entered blinded chart numbers for each site.

For inter-rater reliability, both auditors re-entered data into REDCap from all 100 paired mother-baby charts from the first site. This was done in three stages with comparison and discrepancy resolution after each stage to help auditors learn and improve their process. Once they had completed the first 50 charts, the results were compared and the percent agreement and inter-rater reliability was calculated. The project team then discussed discrepancies and developed a consistent approach for data collection for these elements. The auditors then went back and corrected records where there had been errors and independently audited an additional 20 records. Agreement between the auditors was reassessed, discrepancies were discussed, data captured in REDCap were again corrected and the final group of records was re-abstracted. Auditor agreement on the final portion of records reviewed was above 95% for all data elements abstracted. Outstanding discrepancies between the auditors were discussed and corrected for this final section of data.

Once the agreement between the auditors met our threshold (95%), they began re-abstracting data from the additional sites recruited for the audit (one auditor per chart). While this process was ongoing, a log of changes and anomalies from different sites was created to document changes and deviations from the protocol that occurred.

### Data collection

Data re-abstraction took place from August to November, 2015. The charts (paper or electronic records) were obtained from the Health Records Departments of each of the participating hospitals. The auditors re-abstracted the data into the REDCap data entry forms, which included drop-down menus matching those found in the Registry database’s entry screen. For ease of data entry, the data elements were placed in the same order as they appeared in the majority of hospital records. Data were entered using laptop computers and a secure logon to REDCap.

### Data analysis

We imported the re-abstracted chart data from REDCap into SAS (version 9.4) for analysis, where they were merged with the original data entered into the Registry database. We used percent agreement, Cohen’s kappa statistic (κ) for binary data and intraclass correlation coefficient (ICC) for continuous data [32] to compare the data re-abstracted from patient records with data previously entered into the Registry. We did not impute any values, thus, if data were missing in one or the other source, this was considered a disagreement. If data were clinically relevant and missing in both data sources, then this was considered to be an agreement.

#### Categorical data elements

All categorical/nominal data elements were analyzed using two-way cross tabulations and Cohen’s kappa statistic to examine the proportion of responses in agreement in relation to the proportion of responses that would be expected by chance, given symmetrical marginal distributions [33–35]. Cohen’s kappa statistic represents the proportion of agreements after accounting for chance agreement. Kappa values range from 0 (no agreement) to 1 (total agreement). A kappa value of 0.90, for example, indicates almost perfect agreement while a kappa value of less than 0.60 reflects only moderate agreement [36].

#### Continuous data elements

For continuous data elements, we assessed raw percent agreement using an equal/not equal statement. Additionally, we calculated an ICC which is a more appropriate measure of reliability for continuous data than Pearson’s product moment correlation coefficient or Spearman’s rank-order correlation coefficient since these measure association rather than agreement [33]. ICC values range between 0 (no agreement) and 1 (total agreement) [37]. An ICC over 0.90, for example, indicates excellent agreement, while an ICC less than 0.50 indicates poor agreement between data elements [38]. The notes below Table 1 provide more detailed interpretation of kappa and ICC values.

**Table 1:** Percent agreement, Cohen’s kappa and intra-class correlation coefficient (ICC) for re-absracted data elements

Data Element	Coding	Matched n/927 (%)	Kappa (κ) (%)	95% CI	ICC (%)	95% CI
SITE ID						
Maternal chart ID						
Baby chart ID						
1. Episiotomy^d^	None Medio-lateral Midline Medial Unknown	847 (91.4)	0.67	0.61–0.73		
2. Diabetes and Pregnancy (17 possible pick list choices - MS^b^)	Yes No Unknown	855 (92.2)	0.79	0.72–0.86		
3. Intention to breastfeed	Yes No	757 (81.7)	0.30	0.22–0.37		
4. Newborn feeding at discharge	Formula only Combination Breastmilk only Not applicable Other Unknown	706 (76.2)	0.68	0.64–0.73		
5. Newborn discharged or transferred to^d^	Home Child and family services apprehension Transfer to NICU/SCN other hospital Transfer to NICU/SCN same hospital Transfer to pediatric unit	859 (92.7)	0.46	0.25–0.68		
6. Hypertension during pregnancy	None Eclampsia Gestational hypertension HELLP Preeclampsia Pre-existing hypertension with superimposed preeclampsia Unknown	893 (96.3)	0.58	0.46–0.70		
7. Group B Strep screening results	Done, negative result Done, positive result Result unknown Urine positive for GBS Not done Unknown if screened	739 (79.7)	0.75	0.71–0.79		
8. Group B Strep screening not done reason^a^	Declined screening Other Previous baby with GBS disease Urine positive for GBS	880 (94.9)				
9. Labour type^d^	Spontaneous Induced No labour	908 (98)	0.61	0.57–0.65		
10. Mother resides with cigarette smoker at time of prenatal visit	Yes No Unknown	914 (98.6)	0.68	0.64–0.71		
11. Mother resides with cigarette smoker at time of labour /admission	Yes No Unknown	780 (84.1)	0.67	0.64–0.71		
12. Maternal cigarette smoking at prenatal visit	None <10 /day 10-20 /day >20/day Unknown	835 (90.1)	0.56	0.49–0.62		
13. Maternal cigarette smoking at time of labour^d^	None <10 /day 10-20 /day >20/day Unknown	726 (78.3)	0.58	0.50–0.65		
14. Type of birth^d^	Spontaneous vaginal Assisted vaginal Induced or spontaneous labour CS No labour CS	876 (94.5)	0.89	0.79–1.00		
15. Indications for caesarean section (28 possible pick list choices – MS^b^)^d^	Yes No	901 (97.2)	0.92	0.89–0.95		
16. Maternal Health Conditions (79 possible pick list choices – MS^b^)	Yes No	916 (98.8)	0.75	0.61–0.89		
17. Complications of Pregnancy (24 possible pick list choices – MS^b^)	Yes No	918 (99)	0.79	0.65–0.92		
18. Pain relief measures during newborn screening or serum bilirubin	Breastfeeding Skin to Skin Sucrose Other None Unknown	834 (90)	0.50	0.41–0.59		
19. Labour and birth complications (21 possible pick list choices – MS^b^)	Yes No	913 (98.5)	0.88	0.81–0.94		
20. Indications for induction of labour (23 possible pick list choices – MS^b^)^d^	Yes No	851 (91.8)	0.76	0.70–0.80		
21. Fetal surveillance^d^	Admission EFM strip Auscultation Intrapartum EFM (external) Intrapartum EFM (internal) No monitoring Unknown	922 (99.5)	0.95	0.91–0.99		
22. Number of fetuses^d^	Number (0–8) Unknown	925 (99.8)			0.90	0.89–0.91
23. Number of previous cesarean births ^c, d^	Number (0–6) Unknown	909 (98.1)			0.63	0.59–0.67
24. Maternal pre-pregnancy weight	Weight	887 (95.7)			0.82	0.79–0.84
25. Maternal height	Height	925 (99.8)			0.54	0.50–0.59
26. Maternal weight at end of pregnancy	Weight	924 (99.7)			0.49	0.44–0.55
27. Estimated date of birth	DD-MM-YYY	605 (65.3)				
28. Date of birth	DD-MM-YYY	877 (94.6)				
29. Gestational age at birth^c, d^	Weeks	771 (83.2)				
Gestational age at birth^c^	Days	527 (56.9)				

Notes: ^a^The cell sizes across the response options were too small to run kappas.

^b^*MS* Multiselect

^c^Unable to report ICCs due to lack of convergance of algorithm

^d^Data elements evaluated in the Niday Perinatal Database Re-abstraction Study [24]

Cohen’s kappa statistic (κ) - degrees of agreement after chance agreement has been excluded [36]: Poor < 0; Slight = 0–0.20; Fair = 0.21–0.40; Moderate = 0.41–0.60; Substantial = 0.61–0.80; Almost perfect = 0.81–0.99

Intra-class correlation coefficient (ICC) [38]: Poor < 0.50; Moderate = 0.50–0.75; Good = > 0.75–0.90; Excellent > 0.90

## Results

**Fig. 2:**
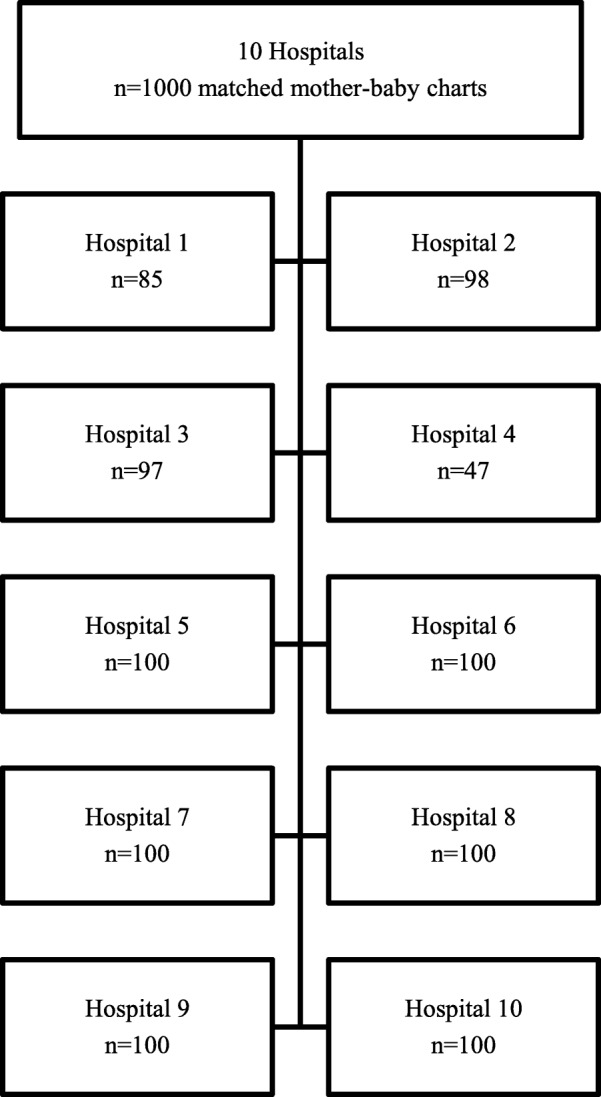
Flow diagram of charts included

Ten hospitals from across Ontario participated: two from the 47 Level 1 hospitals; six from the 41 Level 2 hospitals; and two from the eight Level 3 hospitals. A combination of both paper and electronic documentation systems and a variety of data entry processes were used by the sample hospitals. The total number of charts re-abstracted for this project was 927 linked mother/baby records (Fig. 2). We did not achieve the full target of 1000 charts because some of the requested patient charts were not available during the re-abstraction period.

**Fig. 3:**
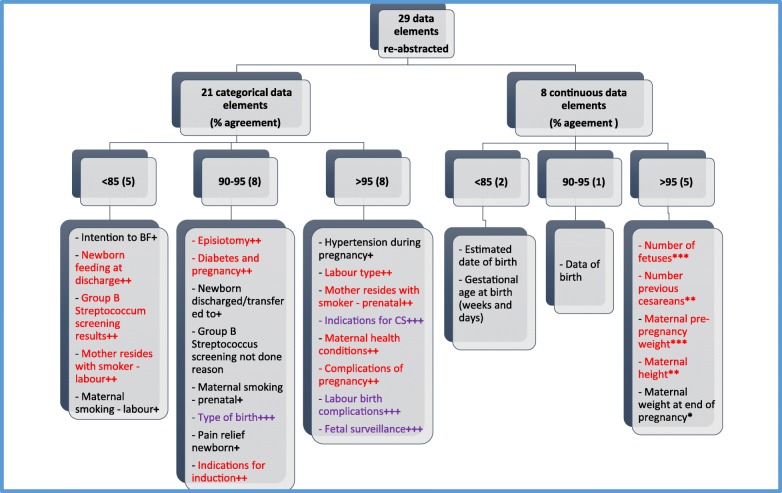
Summary of results (percent agreement). Cohen’s kappa statistic (κ) - degrees of agreement after chance agreement has been excluded (Landis & Koch, 1977): + ≤ 0.60; ++ 0.61–0.80; +++ > 0.80. Intra-class correlation coefficient (ICC) (Portney & Watkins, 2000): * < 0.50; **0.50–0.75; *** > 0.75

A total of 29 data elements were re-abstracted from patient records to assess the degree of agreement with data already in the Registry. The overall results are summarized in Fig. 3 (percent agreements) and Table 1 (percent agreements, Cohen’s kappa or ICC). Of the 29 data elements (21 categorical and 8 continuous) re-abstracted, 22 (75.9%) showed >90% agreement, suggesting that these data elements may be used with confidence.

Of those categorical data elements with >90% agreement, four had kappa values >0.80 indicating almost perfect agreement (beyond chance) (*type of birth, whether there were indications for cesarean section, labour and birth complications, and fetal surveillance type*)*.* Seven categorical data elements had kappa values from 0.61–0.80 demonstrating substantial agreement (beyond chance) (*labour type, mother resides with smoker at first prenatal visit, maternal health conditions, complications of pregnancy, diabetes in pregnancy, episiotomy, and indications for induction)*. There were four categorical data elements with high agreement (> 90%), but kappa values < 0.60 (suggesting agreement could have been due to chance), that warrant further investigation (*hypertension during pregnancy, location newborn discharged/transferred to, maternal smoking at first prenatal visit, and pain relief for the newborn).* Five of the 21 categorial data elements demonstrated < 85% agreement and need further investigation (*intention to breastfeed, newborn feeding at discharge, Group B Streptococcus screening results, mother resides with a smoker at time of labour/admission, and maternal smoking at time of labour/admission).*

Of the eight continuous data elements re-abstracted, six had agreement > 90% (*number of fetuses, number of previous cesarean births, maternal pre-pregnancy weight, maternal height, maternal weight at end of pregnancy*, *and infant’s date of birth*). Of those, four elements had ICC values > 0.50, demonstrating moderate to excellent agreement (beyond chance) (*number of fetuses, maternal pre-pregnancy weight, maternal height, and number of previous cesarean births).* Although there was high agreement (> 90%) for *maternal weight at end of pregnancy,* ICC values were < 0.50 (suggesting agreement may have been due to chance), therefore, this data element warrants further investigation as do the data elements, *estimated date of birth and gestational age at birth (weeks and days),* which were found to have < 90% agreement.

## Discussion

In this data re-abstraction study we found moderate-to-high levels of agreement (beyond chance) between the data collected routinely in the Registry with data collected through this chart re-abstraction. Although neither of the datasets used during the audit can be declared as a gold standard, these results suggest that these core Registry data elements have high validity [39], as do the data elements used to define the key performance indicators in the Maternal Newborn Dashboard [5].

Although reasons for non-agreement were not always apparent, we identified a number of potential contributing factors. These include: discretionary completion of data elements during original data entry (as opposed to a compulsory data element); lack of clarity of information available in the health record; and inaccurate or duplicate documentation in the health record. First, in reviewing the non-agreements and based on feedback from the auditors, it was evident that in some cases the auditor found and entered information from the patient record that the hospital data entry person did not enter. Despite the fact that the goal for both groups was to ensure complete and accurate data entry for each case, in reality, selection of ‘unknown’ during original data entry for some data elements contributed to the non-agreement. Although there are validation checks and missing data reports built into the Registy, along with an extensive monthly data verification process, and BORN Coordinator support for all user organizations, this suggests there is a need for additional initiatives to ensure incomplete records are minimized and that only essential, meaningful data are collected, which would reduce redundancy in this dataset.

The second issue related to the availability of information in the patient health record. If detailed information was not documented in the patient record to match the pick list choices in the Registry, data quality was affected. For example, in the case of *infant pain relief during newborn screening or bilirubin screening,* documentation was not always available to capture this practice in a standardized way creating discrepancies between what was entered in the Registry and what the auditor found in the chart. This example illustrates the critical importance of aligning documentation tools with data entry processes to enhance data quality.

The third issue related to inaccurate or duplicate documentation. Data entry is dependent on the accuracy of the information recorded in the patient record. Even though specific documents were used as the source of information for data entry, some information was difficult to find, or inconsistent, within the patient record, contributing to non-agreement. For example, with *maternal weight at end of pregnancy*, multiple entries of this data with differing values within the patient record may have contributed to non-agreement.

Ten of the data elements included in this re-abstraction study were also evaluated as part of a validation study of the historical perinatal database (i.e., the Niday Perinatal Database audit) [24] (Table 1- see d). We assessed agreement to be consistently above 90% for eight of these data elements in both audits. One of the data elements found to be less reliable during the Niday audit – *maternal smoking at the time of labour/admission* (agreement 78.9% - kappa, 0.51) [24] – was also identified in this audit as requiring further investigation (agreement 78.3% – kappa, 0.58). The data element *episiotomy*, which had 82.7% agreement (kappa, 0.47) in the Niday audit has improved in the new Registry database, with agreement of 91.4% (kappa, 0.67) in the current audit. Other data elements such as: *labour type, type of birth, indications for cesarean section, number of fetuses, and previous cesarean births*, which had very high agreement and kappa values in the Niday audit, remain valid.

The practical contribution of this study is that a subset of data elements has been evaluated for accuracy and comparability with the patient health record, validating them for use by clinicians, policy makers and researchers and identifying potential issues with some data elements that need further exploration by the BORN Data Quality Team. From a Knowledge Translation (KT) perspective the results of this study will increase confidence in the accuracy of the data and build trust in the evidence produced from it.

Although most data elements can be used with confidence, we found a number of data elements to be potentially problematic. The data elements re-abstracted through this audit are all priority items for BORN and its stakeholders, and further investigation of the issues identified will be undertaken by the BORN Data Quality Team to develop strategies to improve the quality of these data elements in the Registry. Ensuring completeness and high validity of the data entered into the Registry and finding ways to enhance data quality are paramount, especially since patient care or funding decisions may be made using administrative or Registry data. The Canadian Institute for Health Information (CIHI) and other clinical registries, are all seeking similar ways to enhance their quality [22, 23, 27]. Based on the results of this audit, and through consultation with experts in the field, a number of recommendations have been identified to improve data quality (Table 2).

**Table 2:** Recommendations to improve data quality

Recommendations to Improve Data Quality	
1. Enhance the data dictionary and data entry guidelines documents to standardize collection and use of data in the Registry;	
2. Clarify definitions (e.g., hypertension during pregnancy, location discharged or transferred to, labour type);	
3. Continue to monitor data quality in each organization;	
4. Communicate with hospital users regularly about data quality issues identified and support corrective strategies to reduce the occurrence of errors;	
5. Create site-specific audit tools for hospitals to monitor their own data quality and identify potentially modifiable data quality issues that could be addressed early;	
6. Continue to encourage accurate documentation in the patient health record to ensure complete information for data entry personnel (e.g., newborn pain relief);	
7. Set automatic verification checks at the time of data entry (e.g., height, weight, gestational age);	
8. Create logic checks where possible based on practice guidelines (e.g., fetal surveillance);	
9. Reassess and refine data element pick list options for problematic data elements (e.g., intention to breastfeed, newborn discharged or transferred to, hypertension during pregnancy, maternal smoking at first prenatal visit and at labour) to align these data elements with the patient health record documentation and optimize data capture;	
10. Provide ongoing training for new staff, to ensure that all data entry personnel are aware of the data elements to be entered, where to find the information and how to address issues of discrepancy when they occur.	

### Limitations

There are limitations to this study. First, the process we used for analysis deviates from some other published data re-abstraction studies [28, 40] insofar as we did not declare the re-abstracted chart data to be the ‘gold standard’. Although sensitivity and specificity can be used to measure the accuracy of data, comparing an external source to a primary source of data requires one of the data sources to be identified as the gold standard [41]. Many factors can affect the quality of data transferred from the patient record, such as observer variation, incomplete or illegible documentation, lack of availability and timeliness of chart completion [42], making it impossible to identify a gold standard from either the original data entered into Registry or the re-abstracted data entered by the auditors. In such cases, when neither data source can be designated as the gold standard, high agreement between the two sources suggests a high degree of validity – a measure of data quality [41, 43].

Although we compared two data sources we cannot definitively conclude that the differences observed between the two are due to inadequacies in the Registry data, as not all data elements collected in the Registry are routinely available in the patient chart. Additionally, some sites have nursing staff enter data into the Registry in real time while providing patient care. Therefore, the person originally entering data may have much more familiarity and depth of knowledge about the clinical scenario than our abstractors. However, as the hospital chart is the official legal medical record for a patient, it should be considered the standard record of care received. From an analytical perspective, this does not influence the analyses we performed on these data; however, it does impact our interpretation of the results and the implications for improvements in the future.

Where there was disagreement and inconsistency between the two data sources, part of this difference could be due to data error in the Registry, erroneous data entered during the audit, or errors in both datasets. Error due to data entered during the audit is likely minimal given the stringent procedures followed. A prevalence effect due to asymmetrical imbalances of marginal totals may have contributed to low kappa values for some of the data elements [44]. These have been flagged for further investigation.

In addition to these larger considerations, the data abstraction tool used for this work could also be a limitation. Care must be taken to ensure the method of data capture for the re-abstracted data does not introduce additional bias. By using a data entry form in REDCap, that allowed for the entry of data only on a single patient and with built in pick list options and range requirements to facilitate accurate data collection, we were able to minimize the introduction of bias by our abstractors. Using REDCap significantly minimized the introduction of errors from our data abstraction process, as compared to using an Excel spreadsheet (as we did in previous pilot work for this process). However, the REDCap abstraction tool did not mimic the actual data entry screens in the Registry, nor, in some sites, the actual flow of data in the patient health record. As a result, the abstractors did find the flow of data entry into our abstraction tool to be a challenge at times. Given the diversity and unstandardized nature of hospital documentation systems across Ontario, it is challenging to design a tool with a data entry flow to match all possible systems. In the future, ensuring a chronological data flow for our entry tool could improve the process and further minimize errors introduced by the abstraction process.

A lesson learned from this work was the need for more rigorous data element definitions in the Registry database. Because data entry processes vary across sites (e.g., clerk-entry, nurse-entry, upload from electronic medical record), there is the potential for different interpretations of the way data are captured and cases are classified. This variability in data entry systems makes it difficult to assess the accuracy of certain data elements, when the source of these data or the way they were recorded varies from site to site. We intend to enhance the data dictionary and data entry guidelines available to maternal-newborn hospitals in Ontario as a deliverable of this project. This will help to facilitate a robust, rigorously developed, and standardized system of data entry across the province.

## Conclusions

The accuracy of most of the data elements included in this study was very good. However, some of the data elements audited need to be strengthened and these issues will be addressed by the BORN Data Quality Team through their work to improve data definitions, enhance training for data entry personnel and review data element revisions and changes through the enhancement process. This study contributes valuable information that will help to improve the quality of BORN data, increasing trust and use of the data to facilitate quality improvement, patient care, and research.

## Data Availability

The data analyzed during this study is held securely at the prescribed registry BORN Ontario. Data sharing regulations prevent this data from being made available publicly due to the personal health information in the datasets. Enquiries regarding BORN data must be directed to BORN Ontario (Science@BORNOntario.ca).

## Abbreviations

BMI: Body Mass Index
BORN: Better Outcomes Registry & Network Ontario
CHEO: Children’s Hospital of Eastern Ontario
CIHI: Canadian Institute for Health Information
ICC: Intra-class Correlation Coefficient
REDCap: Research Electronic Data Capture
